# Use of a Mobile Health Intervention by Older Versus Younger People with HIV: Analysis of Usage, Social Support, and Network Interactions

**DOI:** 10.1089/tmr.2022.0035

**Published:** 2022-12-20

**Authors:** Tabor E. Flickinger, Breanna R. Campbell, Allyson Timm, Sonia Baee, Debajyoti Datta, Sheela V. Shenoi, Julia Rozanova, Rebecca Dillingham

**Affiliations:** ^1^ Department of Medicine, University of Virginia, Charlottesville, Virginia, USA.; ^2^ School of Medicine, University of Virginia, Charlottesville, Virginia, USA.; ^3^ School of Engineering and Applied Science, University of Virginia, Charlottesville, Virginia, USA.; ^4^ Department of Medicine, Yale School of Medicine, New Haven, Connecticut, USA.

**Keywords:** mobile health, digital health, HIV/AIDS, older adults, social support

## Abstract

**Background:**

People with HIV in the United States are aging, with risk for negative health outcomes from social isolation. *PositiveLinks* is a mobile health (mHealth) intervention that includes an anonymous Community Message Board (CMB) for peer-to-peer conversations. We investigated differences in CMB usage and social support between younger (<50 years) and older (≥50) members.

**Methods:**

We assessed the relationship between age groups and app use using chi-square tests. CMB posts were analyzed qualitatively to categorize forms of social support. To have a visual understanding of this relationship, we created a network diagram to display interactions among PL members.

**Results:**

Among 87 participants, 31 (42.5%) were in the older age group. Older members launched the app more often at 6 months (445.5 vs. 240.5 mean launches per participant, *p* ≤ 0.001) and 12 months (712.3 vs. 292.6 launches, *p* ≤ 0.001) compared with younger members. Older members also demonstrated more CMB posts at 6 months (47.4 vs. 7.6 mean posts per participant, *p* = 0.02) and 12 months (77.5 vs. 10.6 posts, *p* = 0.04). Of 1861 CMB posts, 7% sought support and 72% provided support. In addition, the network visualization showed that four participants, who were in the older age group, had more post generation than others and most of their posts provided support.

**Conclusions:**

Older PL members demonstrated significantly more app use than younger members, including CMB posts for social support. This durable app engagement indicates that mHealth can enable social connection among people living with chronic disease across the lifespan.

## Background

The community of people with HIV (PWH) in the United States is aging,^1^ which correlates with more chronic health conditions (multimorbidity) and worsened medical outcomes.^2–5^ Older people with HIV (OPWH) are also at high risk for negative health outcomes related to social isolation.^6,7^

*PositiveLinks* (PL), a tailored mobile health (mHealth) platform to support engagement with HIV care, is utilized by patients across the spectrum of sexual identities, patients living below the federal poverty level, and patients with disability.^8–10^ The PL smartphone app was developed and implemented at the University of Virginia, informed by user-centered design and evidence-based principles of behavioral health and chronic disease self-management.^10^ The app was not commercially available at the time of the study but was subsequently licensed by Warm Health Technologies (WHT), Inc., (Charlottesville, VA) under the name PLCares. The PL platform includes daily self-monitoring of medication adherence, mood and stress, weekly quiz questions, tailored resources, tracking of laboratories and appointments, secure messaging with the clinic team, and an anonymous Community Message Board (CMB). The CMB allows communication between PL users, which improves social connection and support.^11^

Although adults >50 years show interest in health and wellness technology,^12^ they may not have high technological literacy, leading to disparity in benefit from mHealth.^13^ Strategies to facilitate mHealth uptake by older adults include simpler easy-to-use tools, providing information to participants on their individual progress, and encouragement from other participants.^14^ These strategies were used in the development of PL, in collaboration with PWH, including OPWH.^10^ Therefore, we hypothesized that PL would demonstrate usage by OPWH, as well as younger users, and would support social connection and engagement.

We investigated whether older PWH utilized PL as much as our younger cohort and if PL usage among older PWH was durable. We also evaluated the content of CMB posts to determine patterns of seeking and providing social support and investigated the development of social connections in the network of CMB posters.

## Methods

### Setting

CMB data were collected in the context of a prospective cohort study evaluating the usage and impact of PL.^15^ Care providers at the University of Virginia's Ryan White program identified potential study participants as PWH who were “at risk” for disengagement from HIV care, including patients newly initiating or restarting care at the clinic and patients facing challenges such as history of medication nonadherence, missed appointments, or social barriers to care. Participants enrolled on a rolling basis between June 2016 and March 2017 and were followed for 12 months. The clinic serves a population that is predominantly nonurban; >50% over 50 years old; 45% Black; and 40% low-income (<200% of the federal poverty level). The study was approved by our Institutional Review Board (IRB).

At enrollment, participants were trained on smartphone and PL app use. Participants who did not own a smartphone at baseline were provided with one. Participants had access to PL staff for troubleshooting of the smartphone or the app. To offset costs to participants, monthly credits for cell phone service were issued during the study period. Phone credits were issued to participants who met a minimum usage requirement of responding to at least 48% of the daily self-monitoring queries. Participants were aware of this requirement and given feedback on how they were doing on meeting it each month. There was no usage requirement linked to use of the CMB; participants could post as much or as little as they wished without any impact on their phone credits.

### Participant characteristics

Baseline questionnaires were administered verbally by researchers to assess demographic data. Measures of HIV care (CD4 count, viral load, and Health Resources & Services Administration [HRSA-1] retention in care measure^16^) were obtained from participants' medical charts.

### App usage

Measures of participant app usage were collected for 12 months after enrollment from the app server, including response rates to daily queries, count of app launches, and count of CMB posts. The daily queries asked each participant to report whether they had taken their medication and to rate their mood and stress. Participants enrolled in the study moved over to usual care and continued to be PL members with access to the platform, phone credits, and PL staff support after the end of the study.

All CMB posts made during the study enrollment period (June 2016–March 2017) were recorded for textual analysis. Additional CMB posts after the transition of the program to usual care were not collected, so that only conversations between the study protocol participants would be included and not members who joined PL in usual care.

### Qualitative analysis

We examined CMB posts made by participants during the study period using Dedoose, Version 8.0.35 (SocioCulturalResearchConsultants, LLC, Los Angeles, CA). We adapted a codebook from a prior study examining the exchange of social support in PL CMB posts.^11^ The original codebook was created using an open coding strategy and iteratively refined, informed by the Social Support Behavior Code^17^ and other studies of online social support.^18,19^ For this study, the first 2 months of posts were used to establish codebook adaptation, coded independently by three coders and resolved by consensus. After achieving intercoder agreement of 77%, the codebook was applied to the entire data set. Codes were not mutually exclusive.

“Seeking Support” codes included explicit or implied requests for emotional support (including asking for encouragement, praise, and prayers), informational (e.g., asking for data or education), or instrumental support (asking for a tangible good). “Providing Support” codes included counterparts to the “Seeking Support” codes and two additional subcategories: network support (posts recognizing the community) and esteem support (posts affirming the abilities or talents of another poster).

### Statistical analyses

We used chi-square tests for categorical variables and *t*-tests for continuous variables to test for significant differences in baseline characteristics in older versus younger participants. The age of 50 years and above was used as the threshold for older participants, consistent with prior studies in HIV care.^20^ We tested for differences in usage rates of app features between older versus younger participants at 6 months and at 12 months after enrollment. After CMB posts were categorized by support codes, we assessed for differences in frequencies of posts seeking or providing support generated by older versus younger participants. All analyses were conducted using R, Version 4.0.3 (R Foundation for Statistical Computing, Vienna, Austria).

### Network visualization

To investigate how the participants communicated, we created a network diagram of CMB discussions among participants. In this network, we had two types of nodes. The first type was related to each unique participant, the other based on each unique topic of posts. We made a connection between participants' nodes and topic nodes if the participant was involved in discussing that topic. We defined involvement by (1) if the participants created the topic, or (2) if they made any follow-up posts on a given topic. We calculated the size of the nodes and the thickness of the connections based on their frequency in the CMB posts. In addition, we distinguished the participants and the topic nodes by their corresponding age groups and two main topic codes (i.e., Providing Support and Seeking Support), respectively. All analyses were conducted using Python, and D3 JavaScript.

## Results

### Participant characteristics

There were 87 total participants in the study, with 56 participants below 50 years of age (range 18–49) and 31 participants 50 years of age or above (range 50–66). Participants in the older versus younger age groups were similar at baseline (Table 1). Most of the study population identified as male (62%) and as Black non-Hispanic (49%). The older cohort was significantly less likely to own a smartphone at baseline (42% vs. 70%, *p* = 0.02) and less likely to have a data plan (42% vs. 66%, *p* = 0.06). The older cohort was, therefore, more likely to be provided with a smartphone at study enrollment. There were no significant differences between the cohorts on care engagement or viral suppression at baseline.

**Table 1.** Participant Characteristics

	Age <50 ***n*** = 56 (%)	Age ≥50 ***n*** = 31 (%)	*P*
Age, range (IQR)	18–49 (18)	50–66 (5)	0.65
Gender^a^			
Male	33 (59)	21 (68)	
Female	19 (34)	10 (32)	
Race^b^			0.35
Black non-Hispanic	28 (50)	15 (48)	
Hispanic	4 (7)	0 (0)	
White non-Hispanic	15 (27)	8 (26)	
Multiple races, other, or declined to answer	8 (14)	6 (19)	
Educational level			0.29
Did not complete traditional secondary school	18 (34)	12 (39)	
Completed traditional secondary school or beyond	37 (66)	17 (55)	
Income			0.48
<100% FPL	41 (73)	21 (68)	
≥100% FPL	12 (21)	10 (32)	
Insurance status			0.13
Public, as in Medicare or Medicaid	15 (27)	14 (45)	
Private	31 (55)	15 (48)	
None	10 (18)	2 (6)	
Employment status			0.05
Employed outside the home	26 (46)	7 (23)	
Not employed outside the home	28 (50)	23 (74)	
Have phone	44 (79)	22 (71)	0.67
Have smartphone	39 (70)	13 (42)	0.02
Have data plan	37 (66)	13 (42)	0.06
Engagement in care			
Had an HIV medical care visit in the past 6 months	51 (93)	31 (100)	0.32
Currently taking ART	50 (89)	30 (97)	0.22
Baseline HRSA noncompliant^c^	13 (23)	8 (26)	0.99
Compliant	43 (77)	23 (74)	
Baseline CD4 count <200	10 (18)	2 (6)	0.44
200–350	5 (9)	3 (10)	
>350	39 (70)	21 (68)	
Baseline viral load >200	11 (20)	3 (10)	0.49
Suppressed	41 (73)	24 (77)	

Percentages may not add up to 100% due to rounding or missing values.

^a^ Transgender individuals were represented in our study but are not present in the table given potential for identification.

^b^ American Indian/Alaska Native and Asian individuals were represented in our study, but are not present in the table given potential for identification.

^c^ HRSA-1 retention in care measure defined as attendance at two or more appointments, separated by at least 90 days, within the past year.

ART, Antiretroviral therapy; FPL, federal poverty line; HRSA, Health Resources & Services Administration; IQR, interquartile range.

### App usage

**FIG. 1.**
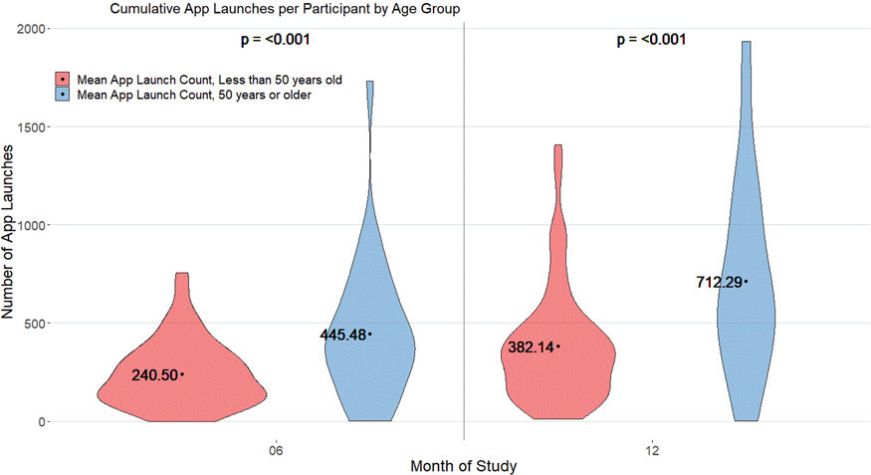
Cumulative app launches per participant among younger (<50-year-old) and older (≥50-year-old) participants.

There was no significant difference in daily query response rate, with both groups responding to >70% of the queries at 6 months. However, there was a trend toward higher query response rate among the older group (67.76% vs. 77.01%, *p* = 0.06). Older members launched the app on average 1.9 times more often than their younger counterparts at both 6 and 12 months (*p* ≤ 0.001; Fig. 1). Also, older members posted on average six times more frequently on the CMB at month 6 (*p* = 0.02) and seven times more frequently at month 12 (*p* = 0.04).

### Qualitative analysis

Of the 1861 CMB posts made by participants, 7% sought support (*n* = 128) and 72% provided support (*n* = 1291), as shown in Table 2. For all quotes, we retained the original spelling and syntax to preserve participant voice. Just under half of participants did not post to the board at all (*n* = 41); three additional participants posted nonsupport messages, such as “small talk” and generic “thank you” posts. Of the remaining 43 participants, 29 individuals sought support and 40 individuals provided support on the CMB. Each age cohort was equally likely to post supportive content on the CMB (50% of the younger cohort vs. 48% of the older cohort, *p* = 1.00). There was a trend in more seeking of informational support by the younger cohort (27% vs. 10%, *p* = 0.11) and provision of informational support by the older cohort (23% vs. 16%, *p* = 0.64), but these relationships did not reach significance.

**Table 2.** Codebook with Examples and Frequencies from the Total 1861 Community Message Board Posts

Support category		Definition	Example	Count (% total)
Seeking support, *n* = 128	Emotional support	Asks for encouragement, comfort, congratulations, praise, empathy, concern, or gratitude. Self-affirmations offered in the virtual community also receive this code.	“Good night my friends. I have a busy day tomorrow and need some prayers. I have a lot of things to do and little time to make it work for me”	86 (5)
	Informational support	Asks for information on a particular subject, including medical or health-related advice, guidance, news, or findings.	“I'M getting low on meds and this is making me feel completely uneasy and sick to my stomach. Anyone else having trouble?”	45 (2)
	Instrumental support	Asks for tangible aid such as contact information, money, food, clothing, products, or goods.	“I'm a male looking for female friend to share apartment and keep each other company and share the bill”	4 (0.2)
Providing support, *n* = 1291	Emotional support	Offers love, concern, humor at no one's expense, or empathy, whether to self or other individual. May be well-wishes, positive maxims, encouragement, prayer, compliments, understanding, and agreement.	“Lol sounds like you had a great time. Thanks for sharing and for your feedback. Have a fantabulos day(emojis)Thanks for the laughter”	1111 (60)
	Network support	Offers sense of belonging to a group of people with similar concerns or experiences. May indicate the presence of the community, the community's unique position to share experiences, availability of community members, and the importance of community closeness. Posts may appear to broaden the intended recipient's social network (e.g., welcome messages).	“Welcome (username) to the PL Family. We are always excited to have new members. Looking forward to getting to know you and be support for you. Please feel free to share at anytime. We will do our very best to help. Please share your knowledge and strength with us. Love and Respect”	400 (21)
	Esteem support	Offers positive assessment of (or confidence in) the intended recipient's abilities, respect for or pride in the intended recipient, or conveys that a particular action/outcome is not their complete fault.	“I'm with someone for 15years.I'm walk away for good because my freedom and happiness.if I can do it so you can. Keep walking and don't look back”	41 (2)
	Informational support	Offers advice or information on a particular subject (medical/health-related information, news, and hobbies). May suggest ways to cope with HIV, share personal experience as an instructive example, teach factual information, refer to another resource, or redefine a situation positively with intention of being helpful.	“CVS takes Randell card or try the pharmacy at UVA if you have patient financial services. If not sign up for it… Let us know how it goes. All is not lost. Also the ppl who make ur meds if you tell them you can't afford sometimes they send you some for free.”	111 (6)
	Instrumental support	Offers goods, services, or performance of a task directly related to the initial post. May include making plans to meet, expressing willingness to help, or actually performing a task or supplying a product/good.	“Roommate you can call me at my number and we can talk about this if you want xxx xxxxxxx”	3 (0.2)

UVA, University of Virginia.

Most posts seeking support were identified as seeking emotional support. In addition to directly asking for encouraging sentiments, participants posted “self-affirmations” to seek emotional support: “Living undetectable, having a baby, getting married… Having HIV will never stop Me from living a fulfilling life.” Three participants responded with congratulations, one of them sharing news of their own. Some participants sought information or advice on specific topics, for example, “Im young. Recently turned 18 and have had HIV for the past 5 months. What are some ways the PL family deals with HIV?” Three participants responded (two of them >50 years old), recommending to “take it a day at a time,” “eventually your gonna have to except the truth WE HAVE hiv and it could be worse…keep asking and keep learning,” and “U just keep on dealing with it… keep your head up and keep moving forward.” Seeking instrumental support was the least common type of support sought.

Much more support was provided than expressly sought on the board, with 10 times as many posts providing support as seeking it. Sixty percent of all CMB posts offered emotional support, whether by encouragement, praise, compliments, prayer, or other uplifting statements. In response to a post introducing a new participant, one individual responded, “Hey [username]… Welcome to the PL Family…We give support and encouragement to one another here. Thanks for your post and no worries no judgement.”

Another young user posted about the loss of a “very good friend,” with four older individuals offering condolences in return. Some interactions addressed difficult topics. One participant posted about a racially charged statement received from an acquaintance, leading to the exchange in Table 3. In this conversation, emotional support is blended with informational support. Although informational support constituted only 6% of the total supportive post content, most questions posed on the board were answered by multiple participants, averaging 2.5 answers for every question.

**Table 3.** An Exchange Between Participants Including Informational and Emotional Support

Confused	
Participant 1 (older group)	So I was talking to a lady and she made a comment like you are very articulate and well mannered for a black woman. I felt offended it feels like a back hand compliment to me. Am I too sensitive? Need feedback family(emojis)
Participant 2 (older group)	You are NOT to sensitive I've been told that but I've learned not to take offense bc that person lacks awareness and that could be bc you just might be the first articulate black person thwy ever spoke to but that is still small minded on her behalf lol
Participant 3 (younger group)	I've had similar interactions with people and I do not take it personally any more. What I've seen is that most people make 90% of there initial judgments based on stereotypes whether they be negative or positive ones. If an adult hasn't learned to judge each person they meet as an individual and not as a representation of the entire group they're being labeled in, then that persons opinions or thoughts really shouldn't matter to you especially if it has anything to do with your self worth. Just be proud of who you are and make sure you never fall into the same mindset as that person who judged who who you where suppose to be just by looking at you.
Participant 1 (older group)	Thanks PL Family for the wisdom and support. Yes I will be mindful of my mindset and biased judgements of others as well. Great feedback and food for thought.(emojis)
Participant 4 (older group)	Well said (participant 3)

Almost one-fourth of all posts (*n* = 400, 21%) offered network support, defined as posts furthering a sense of belonging to the PL community. This included welcoming new members to the board, as in this post: “Hey new members I'm so sorry if I missed greating and welcoming you guys home forgive me but I'm glad you're here.” Participants also shared similarly themed posts with titles such as “roll call” or “shout out.” These messages provided network support to specific members, as in the following example: “Amen very grateful for another day Thanks [username 1] for your post and prayers. Hey [username 2] and [username 3] hope all remains well Have a great day and night and sweet dreams to all members.” Another common form of network support was members sharing positivity with everyone in the “PL family.” For example, “Good morning PL family hope everyone has a great & blessed day” demonstrates both network and emotional support.

Esteem support specifically recognized the abilities and talents of other individuals, often affirming their potential to overcome obstacles. Forty-one posts (2%) received a code for esteem support. In response to a post seeking emotional support, a participant assured the original poster of their capabilities, “I agree 100%. [username] you can make it thru anything. Remember you are not alone cause we here on links are here for you.” Another exchange shared pride and excitement in response to a participant posting good news. “Go [username] I am so proud of you!!! You tried something and looking what happened!!! Smiling from ear to ear. Good Stuff… I am glad you didn't quit 5 minutes before the miracle happene(emojis).”

### Network visualization

**FIG. 2.**
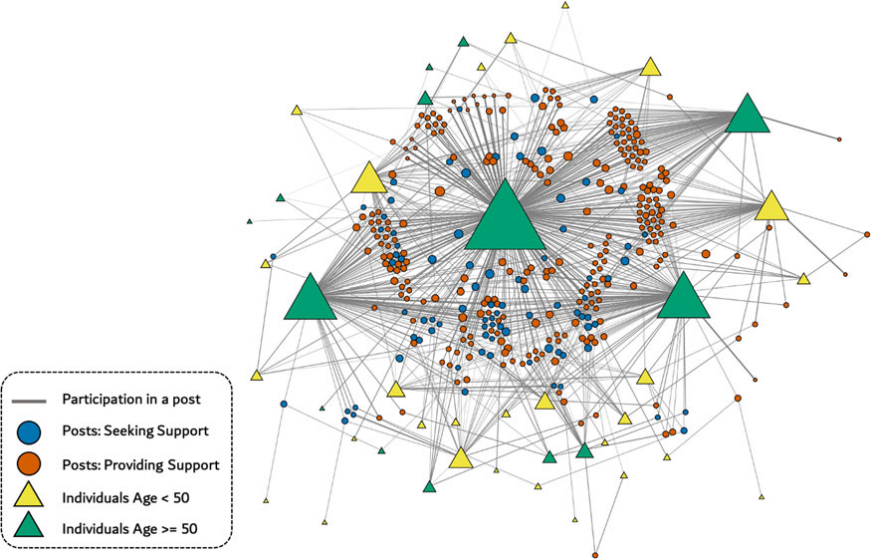
Network diagram depicts interactions by individuals participating in CMB. Size of *triangle* indicates number of interactions. Size of *circle* represents number of messages related to a particular post. The *thickness of each edge* represents the participant frequency of individuals in a given post. CMB, Community Message Board.


The network visualization showed that we had four dominant users. They had *betweenness centrality* >0.1, as shown in Figure 2. The betweenness centrality is a measure of centrality based on the shortest paths. For every pair of vertices in a connected graph, there exists at least one shortest path between the vertices such that either the number of edges that the path passes through (for unweighted graphs) or the sum of the weights of the edges (for weighted graphs) is minimized. Then betweenness centrality of a node *v* is the sum of the fraction of all-pairs shortest paths that pass through *v*:

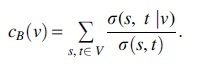


Where *V* is the set of nodes, $\sigma \left(s,t\right)$ is the number of shortest $\left(s,t\right)$ paths, and σ(s,t|v) is the number of those paths passing through some node *v* other than s,t. If s=t $\sigma \left(s,t\right)=1$ and if $v\in s,t,\sigma \left(s,t|v\right)=0$. The nodes with higher betweenness centrality would have more control over the network because more information will pass through that node. Therefore, it means these four participants are important to our communication network since they post more frequently than others in the same cohort. In addition, the color code showed that the posts in the CMB were mainly providing support in comparison with seeking support. This visualization emphasized our finding that older PWH utilized PL more than the younger cohort and interacted with others on the app more often.

## Discussion

We investigated use of the mHealth intervention PL to compare usage patterns of older versus younger PWH. Contrary to common expectations that older people have low uptake of mHealth, we found that older PWH utilized PL significantly more than the younger cohort and interacted with others more often in a durable manner (sustained at 12 months of use). Older PWH were significantly more likely to post on the CMB. Our quantitative analysis revealed differences in usage frequencies, whereas our qualitative analysis allowed us to examine the content. The network visualization depicts the relative participation by age group and the nature of the interactions.

Our observation that older PWH actively used PL at a higher rate than younger participants contrasts with prior reports. Multiple prior studies have documented disparities in accessing online health information, notably that older people use online health information less frequently than younger people.^21,22^ However, older people's willingness and receptivity to technology use have also been shown, as well as benefits of technology in fostering social connection and intergenerational relationships.^23^ Other disparities in online health information usage include higher use by high socioeconomic status groups, women, and White non-Hispanic people.^21^ In contrast, most PL participants identified as Black, male, and living below 100% of the federal poverty level. This suggests PL is accessible for people who may have barriers to using other online health platforms.

Examination of CMB posts demonstrated that support was offered frequently by PL participants, strengthening the community into a “PL Family.” We found no statistically significant differences in supportive posting behavior between age groups. Nonetheless, we observed a trend wherein the younger cohort was more likely to seek informational support, whereas the older cohort was more likely to provide informational support. Overall, emotional support was more prevalent than informational support. This may be because the PL app included other features that offered access to accurate health information, in particular the resources feature and educational quizzes with content curated by the PL team. Participants with specific questions about their own health could also reach out to the clinic team through the secure messaging feature.

The most frequent posters and most prolific providers of support were in the older age group (Fig. 2). This may reflect an important role for intergenerational interaction and an opportunity for PWH with more lived experience to share wisdom and encouragement with younger participants. For OPWH, the community may offer a means of role-modeling or mentoring to assist others who are more recently diagnosed. References to the “PL Family” and welcoming others “home” also suggest a sense of ownership and belonging, which may be especially important for participants who would otherwise be socially isolated.

The private and anonymous nature of the CMB may also help create a safe space for expression. Although loneliness, social isolation, or quality of in-person relationships were not specifically measured in this study, our usability studies of PL have shown, through analysis of interviews with PL users, that the CMB is perceived as a valuable feature for fostering connection and support, while also protecting privacy.^11^ It is not possible to determine from CMB usage whether participants formed online relationships as a substitute for in-person interaction or as a supplement to other sources of support in their lives.

Overall, much of the supportive posting behavior was driven by a select number of participants who belonged to the older cohort. We observed a small group of heavy users, and just under half of participants did not utilize the CMB feature in the study period. A similar phenomenon is described in other digital health social networks, where 1% of users create a majority of content, 9% contribute sparingly, and 90% post little to no content.^24^ Frequent posters facilitate community engagement and provide material to stimulate conversation.^25^ One recent study examined posts that demonstrated leadership in an online HIV support group and found that mentoring and encouragement were the most common leadership types, with participants who had lived with HIV longer providing more leadership.^26^ Online support groups can provide benefit even for more passive users; reading about the experiences of others can be empowering by reducing loneliness and increasing optimism, even for those who do not post.^27^

Prior analyses of PL have shown that engagement in care and viral suppression significantly improve for PL members after 6 months of use and remain significantly improved for long-term members after 24 months.^15^ The original cohort of PL began enrolling in 2013, with secure messaging and other updates added in 2016. The program continued enrolling as a study through 2017, then transitioned from study protocol to usual care. Members with high PL use (responding to at least 48% of daily self-monitoring queries) are more likely than those with low PL use to achieve viral suppression and become engaged in care. Age was not significantly different between members with high versus low PL use and did not appear to influence retention in the PL program.^15^

However, age may play a role in patterns of PL usage when taking multiple features of PL into account. Across the lifespan, different features may have more or different appeal. A latent class analysis of PL usage identified four distinct patterns, based on frequencies of query and quiz responses, CMB posts, and secure messaging with the clinic team.^9^ There was a trend toward age differences between usage groups (*p* = 0.052). Two groups, one with highest use overall (“Maximizers”—median age 48 years) and the other with a higher rate of check-ins (“Check-in users”—median age 50 years) contrasted with “as-needed communicators,” who were younger (median age 37 years) and had lower usage overall.

Limitations to the study include a small sample, which may restrict our ability to demonstrate statistically significant differences. Also, we may have underestimated the amount of support-seeking behavior by posters who desired support when posting but without indicating that desire clearly. Also, study participants were provided with a smartphone if needed, which was important for equitable access to the intervention but may make it difficult to generalize usage patterns observed in study conditions to other populations. OPWH in this study were more likely to have received a new smartphone. The novelty of the technology and/or gratitude associated with receiving the smartphone may have motivated greater engagement with PL.

Participants also had access to the study team for help with technical difficulties related to the app or their phones. We did not track usage of technical support by older versus younger participants. We used the age of 50 years and above to define “older” participants, consistent with prior studies in HIV care,^20^ as opposed to a cutoff of 65 years or other definitions used in other chronic conditions or aging processes. HIV has been associated with premature risk of age-associated comorbidities, such as cardiovascular disease and neurocognitive impairment. However, it is unknown to what extent technology use behaviors may differ between older PWH and other aging adults, so generalizability to other populations must be made with caution.

## Conclusions

Our study shows that OPWH demonstrate durable mHealth use and interact at significantly higher rates than younger individuals, possibly indicating that mHealth enables social connection among older individuals living with chronic disease. PL was developed in collaboration with PWH, including OPWH. Other mHealth interventions may benefit from taking the needs and preferences of older users into account to improve equitable uptake. As OPWH are more likely to have multimorbidity and to be socially isolated, tailored mHealth interventions may be especially important to strengthen connections to accurate health information as well as social connections, supporting health across the lifespan.

## Abbreviations Used

ART: antiretroviral therapy
CMB: Community Message Board
FPL: federal poverty line
HRSA: Health Resources & Services Administration
IRB: Institutional Review Board
IQR: interquartile range
mHealth: mobile health
OPWH: older people with HIV
PWH: people with HIV
UVA: University of Virginia
WHT: Warm Health Technologies
